# Hematopoietic stem cell gene therapy for the treatment of X-linked agammaglobulinemia

**DOI:** 10.1016/j.omtm.2025.101555

**Published:** 2025-08-12

**Authors:** Christopher R. Luthers, Annika Mittelhauser, Aurelien Colamartino, Xiaomeng Wu, Samuel Cirigliano, Joseph D. Long, Julie M. Sanchez, Zulema Romero, Donald B. Kohn

**Affiliations:** 1 Molecular Biology Interdepartmental Program, University of California, Los Angeles, Los Angeles, CA 90095, USA; 2 Department of Microbiology, Immunology and Molecular Genetics, University of California, Los Angeles, Los Angeles, CA 90095, USA; 3 Department of Pediatrics, David Geffen School of Medicine, University of California, Los Angeles, Los Angeles, CA 90095, USA

**Keywords:** X-linked agammaglobulinemia, Bruton’s tyrosine kinase, hematopoietic stem cells, gene therapy, site-specific genome editing, CRISPR-Cas9, adeno-associated virus, B cell development, immunoglobulins, immunodeficiency

## Abstract

X-linked agammaglobulinemia (XLA) is a rare inborn error of immunity caused by loss-of-function mutations in the gene encoding Bruton’s tyrosine kinase (BTK). XLA patients lack mature B cells and have negligible antibody levels, leaving them susceptible to recurrent bacterial and chronic viral infections. Autologous hematopoietic stem cell gene therapy with gene-corrected HSC may serve as a promising treatment of XLA; this therapy would provide a one-time cure and would replace lifelong immunoglobulin replacement therapy. Due to the requirement of strict physiological regulation of *BTK* gene expression, a site-specific editing strategy was designed to insert a *BTK* cDNA transgene directly into its endogenous locus. To study the effectiveness of this therapy, murine lineage-negative hematopoietic cells from a murine model of XLA were edited using CRISPR-Cas9/rAAV6 then transplanted into recipient XLA mice. Myeloablated XLA mice that received transplantation of Btk-corrected Lin- cells displayed high levels of engraftment, significant increases in their B cell levels, increased production of various immunoglobulins, improved B cell development in the bone marrow, increased B cell receptor diversity, and the ability to produce antigen-specific antibodies following immunization. Collectively, we have modeled a gene therapy strategy in a disease model of XLA and extensively validated the site-specific genome editing approach.

## Graphical abstract

**Figure:**
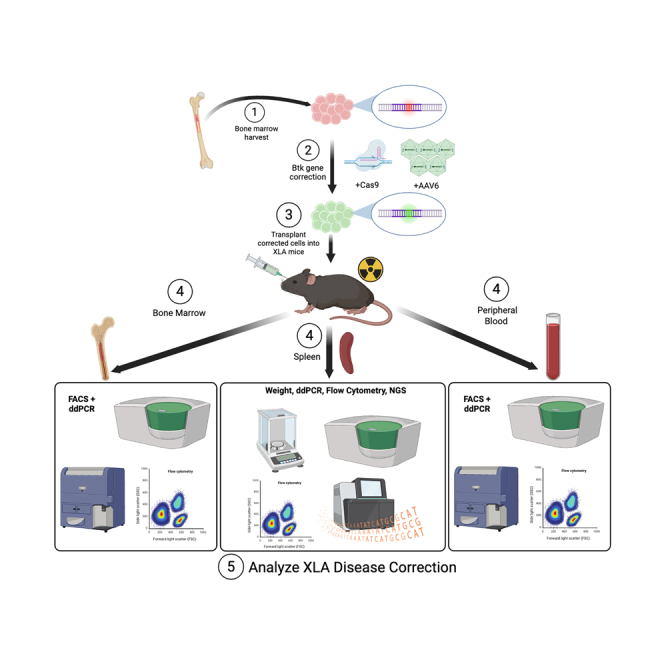

## Teaser

In this study, Kohn and colleagues validated a Btk site-specific genome editing strategy in a murine model of X-linked agammaglobulinemia (XLA) that resulted in restoration of B cell development, antibody production, and response to immunization, paving the way for preclinical studies of a gene therapy for XLA.

## Introduction

X-linked agammaglobulinemia (XLA) is a rare inborn error of immunity caused by loss-of-function mutations in the gene encoding Bruton’s tyrosine kinase (*BTK*). The disease follows an X-linked recessive inheritance pattern and only affects males, with a prevalence of about 1 in 200,000 live births, or 1 in 100,00 males.^1^ BTK is a non-receptor tyrosine kinase of the Tec kinase family and is essential for B cell receptor (BCR) signaling, and early progenitor B cell survival, proliferation, and differentiation.^2^ Defective BTK activity results in a block in the maturation of B cells in the bone marrow of XLA patients, leading to severely reduced or absent peripheral B cells and severe hypogammaglobulinemia.^3^^,^^4^ XLA results in patients being vulnerable to recurrent bacterial and chronic sino-pulmonary infections by bacteria. Early treatment and diagnosis with immunoglobulin replacement therapy have dramatically improved outcomes in XLA patients, but patients continue to be at risk for recurrent pulmonary disease and immune-related complications.^5^

Although BTK is essential for normal immune function, its dysregulation has been implicated in oncogenesis and autoimmunity. Overexpression or constitutive activation of BTK has been implicated in B cell cancers such as CLL and MCL.^6^ The covalent BTK inhibitor ibrutinib, a small-molecule inhibitor, has been found to be efficient against B cell cancers by inhibiting BCR signaling, thus suppressing the proliferation and survival of cancer cells.^7^ Collectively, these observations emphasize the critical importance of strict physiological regulation of BTK expression to prevent both immunodeficiency and carcinogenesis.

Because XLA is a monogenic disorder, correction of the singular *BTK* gene should result in disease correction of agammaglobulinemia, opening a promising avenue for a hematopoietic stem cell gene therapy approach to either add or restore BTK protein expression. Traditional gene therapy approaches, such as lentiviral vector-transduced BTK expression, raise concerns associated with unregulated expression levels and potential insertional mutagenesis.^8^ Lentiviral vectors insert into the genome semi-randomly, increasing the risk of deleterious effects if BTK is expressed at non-physiological levels or in inappropriate cell types, disrupting signaling pathways, and heightening the likelihood of cancer development.^9^

Conversely, the precise correction of genes would maintain endogenous expression of BTK, conserving its original transcriptional and post-transcriptional regulatory mechanisms. A site-specific genome editing approach allows for maintenance of BTK levels within physiological ranges, thus reducing the oncogenic hazards linked to overexpression.^10^ Another major benefit is that a site-specific genome editing approach would be effective independent of the *BTK* mutation, offering a sustainable, mutation-independent therapeutic alternative. XLA may be caused by over 600 individual mutations distributed throughout the entire coding region of the gene that include missense, nonsense, frameshift, and splice-site mutations.^11^ Adding a correct version of the wild-type *BTK* cDNA early in the gene would correct almost all mutations, as insertion of the *BTK* cDNA would prevent transcription of downstream mutations.

One major advantage in the design of an XLA gene therapy is the selective advantage that is provided to BTK-replete cells in splenic environments. Studies have demonstrated that B cell progenitors that correct for BTK deficiency have a competitive advantage in B cell niches, especially in the spleen.^12^ The lack of mature B cells in XLA patients provides an empty niche, which enables even a small number of corrected cells to expand and repopulate the peripheral B cell compartments. This phenomenon underlies the immense therapeutic promise of genome repair strategies, as even a small proportion of precisely edited cells may have a clinically relevant advantage in the long term.^13^^,^^14^

In this study, we describe a genome editing strategy for the correction of *Btk* mutations in a murine model of XLA. Previous similar studies have been conducted validating the feasibility of a *BTK* site-specific editing approach in human cells^15^^,^^16^ using a cDNA donor with regulatory elements discovered in prior studies from our group.^16^ This present report, however, focuses not only on site-specific editing the *Btk* locus, but also the resulting disease correction as evidenced by improved B cell development and antibody production in a murine XLA model. We discuss the molecular design of the gene editing strategy, its ability to restore Btk function in a murine model, and its future application in the clinical setting. Using precise genome editing to reestablish normal Btk function, our goal is to create a therapeutic strategy that is both safe and effective.

## Results

### Validation of a *Btk* site-specific gene editing approach

**Figure 1:**
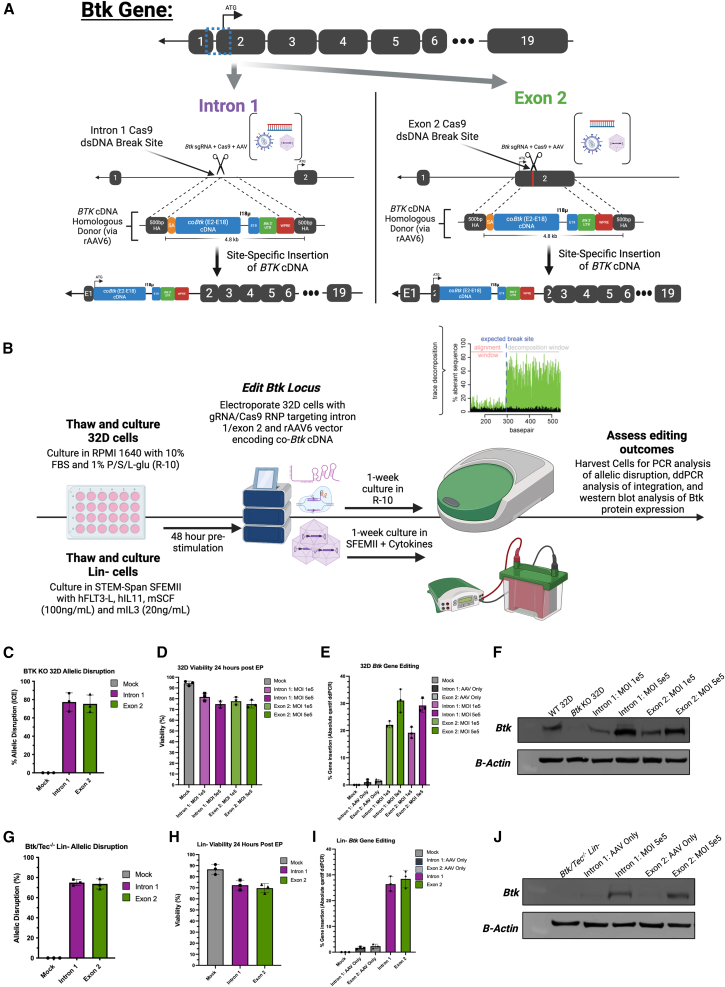
Validation of a Btk site-specific gene editing approach (A) Schematic of *Btk* editing strategy. The top row shows the Btk gene with exons and introns. In the second row, zooming in on intron 1 and exon 2 of the *Btk* gene, shows the sites where sgRNAs targeting these regions will allow for Cas9 dsDNA breaks. In the third row, recombinant adeno-associated virus (rAAV) vectors containing a nearly full-length human *BTK* cDNA with 500-bp homology arms directly flanking the dsDNA break site to allow for homologous recombination at either the intron 1 or exon 2 sites. The *BTK* cDNA donor also contains a “micro” version of the terminal intron 18, *Btk* 3′ UTR, and WPRE element. The bottom row depicts the *BTK* minigene inserted into the endogenous murine *Btk* locus at the intron 1 or exon 2 sites. (B) Outline displaying 32D and Lin- cell editing timeline. Cells are cultured in either R-10 or SFEM + cytokines for the times listed, then electroporated with Cas9 RNP followed directly by transduction with rAAV6 donor. One week post editing, genomic DNA and total protein lysates are harvested for analysis of allelic disruption, gene integration, and exogenous Btk protein expression. (C and G) Genomic DNA harvested from 32D and Lin- cells, respectively, was PCR amplified with primers flanking the Cas9/sgRNA cut sites and sent for Sanger sequencing analysis of *Btk* allelic disruption via synthego ICE analysis. The *y* axis represents the frequency of amplified DNA copies which contained insertions/deletions at the *Btk* dsDNA break site. *n* = 3. (D and H) Twenty-four hours post electroporation, 32D and Lin- cells, respectively, were analyzed for viable cell counts using hemacytometers and trypan blue exclusion to determine the acute toxic effects of editing reagents. The *y* axis represents the average percentage of live cells counted across two individual aliquots of a given sample. (E and I) Genomic DNA harvested from 32D and Lin- cells, respectively, underwent in/out ddPCR analysis using primers and probes that allow for specific quantification of *BTK* cDNA insertion at its endogenous locus to quantify *Btk* site-specific integration frequency. The *y* axis represents the frequency of site-specific *Btk* integration events normalized to a reference housekeeping gene. *n* = 3. (F and J) Western blot protein analysis of Btk expression from Btk^−/−^ 32D cells and Btk/Tec^−/−^ Lin- cells, respectively. Cells were lysed using RIPA lysis buffer followed by western blotting and chemiluminescent detection of Btk and Beta-Actin protein expression levels; 32D cells were transfected with equivalent Cas9-RNPs followed by transduction of rAAV6 at MOIs of either 1e5 or 5e5. To determine the role of rAAV6 transduction alone on exogenous Btk expression, Lin- cells either received Cas9-RNP or no electroporation, followed by transduction with rAAV6 at an MOI of 5e5. *n* = 3.

The site-specific editing approach we used requires a double-stranded break in the genomic DNA (gDNA) of the edited cells accompanied by delivery of a homologous donor sequence encoding the wild-type (WT) human *BTK* cDNA, which has been proven effective in rescuing Btk deficiencies in murine models. To edit the cells, we used a Cas9-RNP targeting either intron 1 or exon 2 of the murine *Btk* gene followed by recombinant adeno-associated virus type 6 (rAAV6)-mediated delivery of a nearly full-length, corrective WT *BTK* cDNA sequence with 500-base pair (bp) homology arms matching the DNA sequences directly downstream and upstream of the Cas9 cut site to allow for base-perfect homologous recombination (Figure 1A).^17^ The start codon for the *Btk* gene is in exon 2, with exon 1 being untranslated^18^; thus intron 1 and exon 2 target sites were chosen to maintain integrity of regulatory elements near the transcriptional-start site. The *BTK* cDNA homologous donor sequence used contains a “micro” terminal intron 18, and the woodchuck post-transcriptional regulatory element (WPRE) to boost expression and protect the nascent *BTK* mRNA upon nuclear export and translation.^19^^,^^20^ The intron 1 donor contains the entire *Btk* reading frame, including the splice-acceptor region of intron 1 to allow for proper splicing, with the exon 2 donor containing a nearly full-length copy of the *Btk* reading frame (Figure 1A). In a previously published study these donors were optimized and empirically validated in human cell lines and primary cells, displaying increased BTK expression per integration event and high levels of gene insertion.^16^

To validate the feasibility of the aforementioned gene therapy approach in murine cells, both Btk^−/−^ 32D murine myeloblast cell lines and Btk/Tec^−/−^ murine primary lineage-depleted “Lin-” cells were cultured and edited with Cas9-RNP + rAAV6. During the initial editing experiments conducted, cells were edited using an inefficiently transducing rAAV6 donor and less effective transduction method in which electroporated cells were placed into 24-well plates overnight in ∼500 μL of medium, cells, and rAAV6. While this method resulted in high levels of Cas9-mediated double-stranded DNA (dsDNA) breaks (Figure S1A), both the viability and integration frequency of the edited cells were quite low (Figures S1B and S1C), raising concerns about whether these editing rates would be sufficient to result in disease correction.

To address this low HDR efficiency, two major alternations were made. First, following electroporation, cells underwent a lower volume transduction in which they were incubated with rAAV6 and medium in a total of 150 μL for 2 hours to allow for initial viral entry, followed by transfer to a larger volume overnight (Figure S1D). The second major change was based on the finding that different rAAV6 manufacturer’s preps can significantly alter cellular health, potential, and site-specific editing efficiency.^21^ Based on these findings, an alternative rAAV6 manufacturer was chosen, which, combined with the lower volume transduction, significantly improved integration efficiency (Figure S1E). Twenty-four hours post electroporation (EP), acute toxicity of editing reagents was analyzed via viability analysis with trypan blue exclusion. Cells were then washed and cultured for multiple days before harvesting gDNA and protein lysates to measure allelic disruption and site-specific gene integration (Figure 1B).

Btk^−/−^ 32D immortalized cells were tested at an rAAV6 MOI of both 1e5 and 5e5 and showed ∼75% allelic disruption at the Cas9 dsDNA break site (Figure 1C), ∼75% viable cells 24-h post-EP (Figure 1D), and 20%–35% *Btk* site-specific gene insertion as measured by droplet digital PCR (ddPCR) (Figure 1E) for both intron 1 and exon 2 targeting loci. Seven days post-EP, protein lysates were harvested followed by western blot analysis of Btk protein expression. High levels of resultant Btk expression were displayed in Btk^−/−^ 32D cells transduced with both intron 1 and exon 2 vectors (Figure 1F).

To validate the site-specific editing strategy in the more relevant cell line used for our murine transplants, primary murine Btk/Tec^−/−^ Lin- cells edited with Cas9/RNP and an rAAV6 *BTK* donor at MOI of 5e5 displayed ∼75% allelic disruption (Figure 1G), ∼70% viable cells 24-h post-EP (Figure 1H), and 25%–30% *Btk* site-specific gene insertion as measured by ddPCR (Figure 1I). Btk/Tec^−/−^ Lin- cells also underwent protein extraction followed by western blot analysis and displayed high levels of resultant Btk expression with both intron 1 and exon 2 rAAV vectors (Figure 1J). Collectively, we were able to successfully design, test, and validate a Cas-RNP + rAAV6 genome editing strategy for the site-specific insertion of *BTK* to predicted therapeutic levels in immortalized and primary murine Btk-deficient immune cells. However, this then raises a new question of whether the editing rates seen are high enough to result in disease correction of XLA phenotypes.

### Btk/Tec^−/−^ Lin- cells displayed high levels of multi-tissue engraftment and maintained *Btk* gene correction in a murine model of XLA

**Figure 2:**
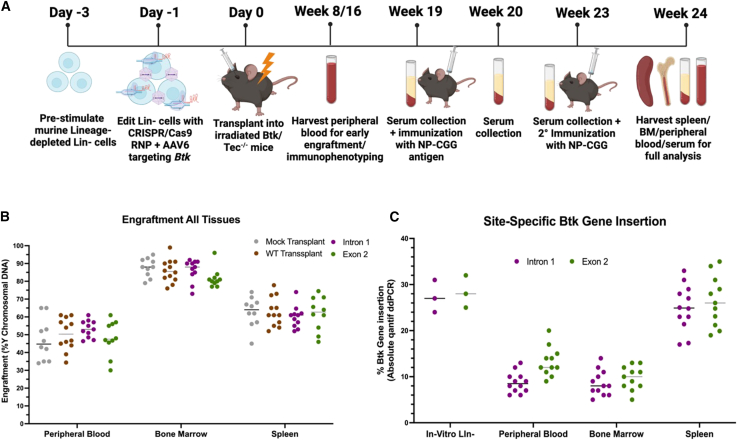
Btk/Tec^−/−^ Lin- cells display high levels of multi-tissue engraftment and maintained *Btk* gene correction in a murine model of XLA (A) Timeline of Lin- cell culture, editing, and Btk/Tec^−/−^ mouse transplantation. Days and weeks are relative to the day of transplant. (B) Engraftment of male donor cells into female recipient mice was measured via ddPCR quantification of male (Y chromosome) DNA as normalized to an input DNA housekeeping control for total DNA harvested from peripheral blood, bone marrow (BM), and spleen of all transplanted mice. The *y* axis represents percent engraftment out of total DNA. (C) Site-specific *Btk* gene insertion was measured via ddPCR analysis using primers and probes that allow for specific quantification of *BTK* cDNA insertion at its endogenous locus, normalized to an input DNA housekeeping control for total DNA harvested *in vitro* input Lin- cells, peripheral blood, bone marrow (BM), and spleen of intron 1 and exon 2 edited mice. *n* = 10, “mock transplant”: Btk/Tec^−/−^ Lin- cells transplanted into Btk/Tec^−/−^ mice. “WT transplant”: WT C57/Bl6 Lin- cells transplanted into Btk/Tec^−/−^ mice. “Intron 1”: Btk/Tec^−/−^ Lin- cells edited with Cas9-RNP + rAAV6 targeting intron 1 transplanted into Btk/Tec^−/−^ mice, “Exon 2”: Btk/Tec^−/−^ Lin- cells edited with Cas9-RNP + rAAV6 targeting exon 2 transplanted into Btk/Tec^−/−^ mice.

To validate the potential for our genome correction strategy to result in therapeutically effective disease correction levels, the gene therapy course for XLA patients was closely mimicked in a murine model of XLA. The XLA mouse model has a complete knockout of Btk and Tec, another Tec family kinase to generate a near phenocopy of human XLA.^22^ Lin- cells from Btk/Tec^−/−^ male mice were pre-stimulated for 48 h to allow for cycling of stem cells prior to editing with Cas9-RNP + rAAV6,^23^ followed by overnight culture and transplantation into myeloablated recipient XLA female mice. Following transplantation, peripheral blood was collected at early time points to confirm engraftment of donor cells to justify continuing experimentation. During the final 5 weeks of the experiment, serum was collected, and mice received primary and secondary immunizations with 4-Hydroxy-3-nitrophenylacetyl Chicken-Gamma-Globulin (NP-CGG), a well characterized T-dependent antigen^24^ followed by final harvest of spleen, bone marrow (BM), peripheral blood, and serum for endpoint analyses. Importantly, the final harvest did not take place until 24 weeks post-transplant, a sufficient period to increase confidence that the phenotypic correction seen was due to true long-term stem cells and not long-lasting progenitors^25^; however, secondary transplants would likely be required to confirm this (Figure 2A). Donor male XLA Lin- cells were transplanted into myeloablated female recipient XLA mice as the sole genotypic difference between the donor and recipient cells in the Y chromosome. ddPCR analysis of the y chromosome DNA copies were normalized to a reference gene to quantify the engraftment rates in peripheral blood, BM, and spleen of all mice.

Transplanted mice displayed high levels of engraftment of donor cells in various immune tissues, with the highest rates (∼80%) in the BM, the truest indicator of stem cell engraftment^26^ (Figure 2B). Genomic DNA from immune tissues then underwent ddPCR analysis of site-specific *Btk* gene correction in various immune tissues. Although the editing rates of the input Lin- cells recipient mice received being ∼25%–30%, this number dropped significantly in the peripheral blood and BM (Figure 2C), a common phenomenon seen in genome editing Lin- cells transplanted into murine models.^27^^,^^28^ Despite this, the genome correction rates in the spleen of recipient XLA mice were markedly higher, closer to ∼25%. This highlights the selective advantage of Btk-replete cells—despite low levels of *Btk* correction in the bone marrow, B-lymphoid precursors can leave the bone marrow and traffic to the spleen where they are able to undergo proliferation, maturation, and expansion, giving rise to the increased quantity of *Btk* gene correction in this tissue. These experiments have successfully validated the ability of this gene therapy approach to allow for engraftment of *Btk* gene-edited Lin- cells into a murine model of XLA, but they do not answer the question of whether this results in correction of XLA disease phenotypes.

### Transplantation of *Btk* genome-corrected cells relieves developmental block and increases production of immature and mature B cells in bone marrow of the XLA mouse model

Btk is a receptor tyrosine kinase in B cells that allows for proliferation, development, and maturation of B cells. Loss-of-function mutations in the *Btk* gene results in a developmental block specifically in the transition from pre/pro B cells to immature/mature B cells.^29^^,^^30^ An effective gene therapy strategy for the correction of XLA would result in a relief of the developmental block in the bone marrow of XLA mice—allowing for a detectable increase in immature/mature B cells. To measure this, total cells from the bone marrow of XLA and WT mice were harvested, counted, and analyzed via flow cytometry to quantify the fractions of B cell development (Figure S2).^31^^,^^32^

**Figure 3:**
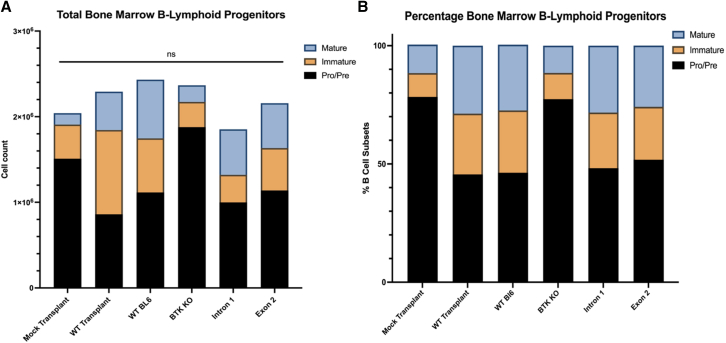
Transplantation of *Btk* genome-corrected cells relieves developmental block and increases production of immature and mature B cells in the bone marrow of an XLA mouse model (A) Total cell counts were determined from single-cell suspensions of mouse whole bone marrow, and populations of each B-lymphoid progenitor were calculated by multiplying percentages of each B-lymphoid subset by total cell counts. Pro/Pre B cells: IgM−, IgD−, B220+; Immature B cells: IgM+, IgD−, B220^+^; Mature B cells: IgM+, IgD+, B220+. The *y* axis represents total cell counts. (B) Percentages of B-lymphoid progenitors were calculated by determining counts of individual B cell subsets divided by total number of all B-lymphoid progenitors combined. The *y* axis represents percentages of individual subsets out of all total B cell progenitors. *n* = 10.

Despite seeing no significant differences in the total number of B-progenitors in the bone marrow of XLA mice (Figure 3A), there were significant increases in the total percentage of immature/mature B cells in the bone marrow of XLA edited mice relative to their transplantation and irradiation controls (Figure 3B) (Table S1). These results not only display an increase in the percentage of immature and mature B cells being produced in the bone marrow of XLA edited mice, but they also provide insight as to specifically where this developmental block is occurring in the absence of functional Btk.

### Transplantation of *Btk* genome-corrected cells increases B cell production and BCR diversity in the spleen of transplanted XLA recipient mice

**Figure 4:**
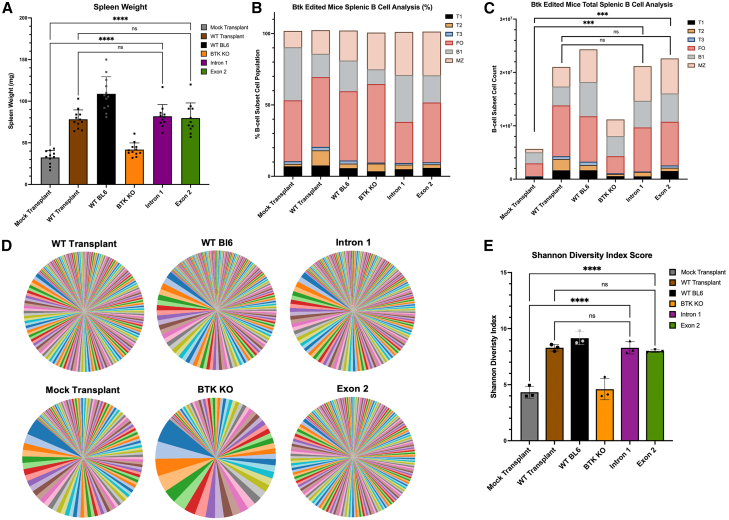
Transplantation of *Btk* genome-corrected cells increases B cell production and BCR diversity in the spleen of transplanted XLA recipient mice (A) Following harvest, spleens were weighed using an analytical balance. The *y* axis represents spleen weight in mg. *n* = 10. (B) Percentages of splenic B cell subsets were calculated by determining counts of individual B cell subsets divided by total number of all B cell subsets combined. The *y* axis represents percentages of individual subsets out of all total B cell subsets. T1: B220+, CD93+, CD23−, IgM^Hi^; T2: B220+, CD93+, CD23+, IgM^Hi^, CD21^Lo^; T3: B220+, CD93+, CD23+, IgM^Lo^, CD21^Lo^; FO: B220+, CD93−, CD23+, IgM+, CD21+’; B1: B220+, CD93-, CD23-, IgM^Hi^, CD21^Lo^; MZ: B220+, CD93−, CD23−, IgM^Hi^, CD21^Hi^. *n* = 10. MZ, marginal-zone B cells; FO, follicular-zone B cells. (C) Total splenic B cell counts were counted from single-cell suspensions of mouse whole spleen, and populations of each B cell subset calculated by multiplying percentages of each B cell subset by total spleen cell counts. The *y* axis represents total cell counts. *n* = 10. (D) Pie charts displaying the top 500 unique BCR sequences by read frequency. Size of each slice correlates to frequency of each sequence. (E) Using the same data used to generate the plots in (D), Shannon diversity index scores for each individual condition were determined from all total unique VDJ sequences and their frequency using a custom script via R-studio. The *y* axis represents Shannon diversity index score. *n* = 3. See “quantification and statistical analysis” section for explanation of *p* values.

The spleen serves as the primary lymphoid organ for B cells and is critical for their proliferation, maturation, and function.^33^ Previous studies have shown that even low numbers of corrected B cells in the BM of Btk^−/−^ mice results in significant increases in the population of *Btk*-corrected cells in the spleens of these mice.^13^ Based on this, an effective XLA therapy should result in detectable increases in the total number, maturation, and diversity within the B cell populations of a recipient. To quantify this, we began by harvesting the spleens of XLA recipient mice, followed by measurement of the total weight of each spleen. XLA mice that were transplanted with Lin- cells edited by either intron 1 or exon 2 *Btk* correction strategies showed significantly higher spleen weights than the mock transplant control (XLA mice that received transplantation of unedited XLA Lin- cells) and no significant difference compared with the positive WT transplant control (XLA mice that received unedited WT C57/Bl6 Lin- cells) (Figure 4A). Spleens were then harvested and total cells were counted and analyzed via flow cytometry analysis of various B cell maturation subsets (Figure S3).^34^

Despite not seeing significant differences in the percentages of the various B cell subsets in the spleen of XLA mice (Figure 4B) (Table S2), the intron 1 and exon 2 edited conditions showed significant increases in the total number of B cells in the spleens compared with the mock transplant control, with no significant alterations relative to the WT transplant control condition (Figure 4C). Although similar levels of engraftment (percentage donor cells) are measured across all transplant conditions (Figure 2B), the significant increase in the total number of B cells in the spleens of WT transplant, intron 1, and exon 2 mice (Figure 4C) indicate significantly higher total number of engrafted-splenic B cells. Interestingly, the total numbers of B cells in the spleens correlated directly with the weights of the spleens, hinting that a major factor in the increase in splenic weight was an increase in the outgrowth of B cells.

The major factor that contributes to the ability of the humoral immune system to fight infections is a broad B cell receptor (BCR) repertoire, which allows for a large diversity of both BCRs and secreted antibodies in the periphery of individuals.^35^ Diversity of BCRs is generated by recombination of V, D, and J regions in the immunoglobulin (Ig)H locus, allowing for heterogeneous Fab regions capable of responding to a broad variety of antigens.^36^ We hypothesized that the increased number of total B cells detected in the spleen would also correlate with an increase in BCR diversity within this cell population. RNA was harvested from splenic tissue followed by reverse transcription and next-generation sequencing (NGS) analysis of the diversity within the VDJ recombinants of the IgH gene domain. The intron 1 and exon 2 edited conditions showed significant increases in diversity relative to the mock transplant control condition, as displayed visually in Figure 4D.

Additionally, results from the NGS analysis were analyzed quantitatively, demonstrating that the intron 1 and exon 2 conditions had a significantly higher Shannon-Wiener diversity index score (which quantifies both the “richness” and “diversity” of a given species)^37^ than the mock transplant control group, with no significant difference compared with the WT transplant condition (Figure 4E). Collectively, mice that received Btk-corrected Lin- cell transplantation showed overall increases in the total number and diversity of B cells in the spleen, giving further validation of the selective advantage of Btk-replete cells in the spleen.

### Transplantation of *Btk* genome-corrected cells increases peripheral blood B cell maturation, total antibody production, and humoral response to immunization

The periphery represents an important environment for all immune cells, making it an ideal tissue for overall immune reconstitution analysis. As previously discussed, one of the major advantages of a site-specific genome editing approach is that lineage-specific expression of the Btk transgene should be maintained in the appropriate cell types, mimicking the endogenous Btk expression pattern. Because of the role of Btk as a tyrosine kinase involved in signal transduction, improper Btk expression in undesired cell types could result in negative consequences such as improper signaling and proliferation, with evidence of this potentially even resulting in the generation of autoreactive T cells.^38^ Additionally, in a parallel study testing *in vitro* editing of human *BTK* led to aberrant BTK expression in a human T cell line.^16^ With this concern, it is critical that a genome editing strategy does not affect proliferation or frequency of any non-desired immune cells and that the Btk lineage expression pattern is maintained similarly to endogenous levels.

**Figure 5:**
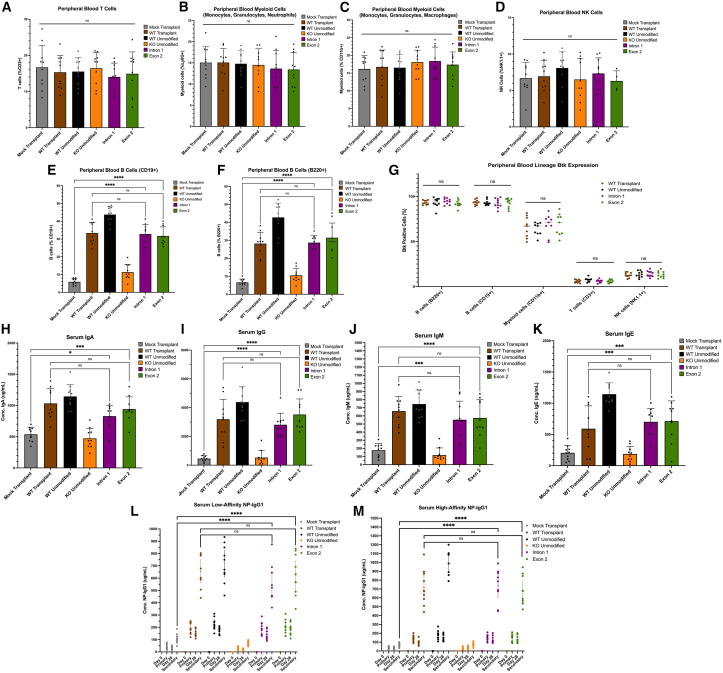
Transplantation of *Btk* genome-corrected cells and increases peripheral blood B cell maturation, total antibody production, and humoral response to immunization (A–F) Frequencies of all immune cell types were calculated via flow cytometry analysis from peripheral blood. T cells: CD45+, CD3+; Myeloid cells: CD45+, Ly6G/C+, Myeloid cells (2): CD45+, Cd11b+; NK cells: CD45+, NK1.1+; B cells: CD45+, CD19+; B cells: CF45+, B220. The *y* axis represents the frequency of given immune cell type listed. *n* = 10. (G) In addition to extracellular staining to identify cell type, intracellular Btk expression was quantified by flow cytometry across all conditions in each cell subtype. The *y* axis represents percentage of Btk-positive cells within the specified cell type. *n* = 10. (H–K) Serum was isolated from peripheral blood followed by ELISA analysis of total IgA, IgG, IgM, and IgE of blood immunoglobulin levels. The *y* axis represents total immunoglobulin levels – note the *y* axis labels for units of measurement per immunoglobulin. (L) Serum was isolated from peripheral blood followed by ELISA analysis of low-affinity anti-NP antibodies in response to NP(40)-CGG immunization. “Low-affinity” anti-NP antibodies are defined as antibodies that are able to bind an ELISA plate coated with NP(4)-BSA. *n* = 10. (M) Serum was isolated from peripheral blood followed by ELISA analysis of high-affinity anti-NP antibodies in response to NP(40)-CGG immunization. “High-affinity” anti-NP antibodies are defined as antibodies that are able to bind an ELISA plate coated with NP(40)-BSA. *n* = 10. See “quantification and statistical analysis” section for explanation of *p* values.

To confirm this, we performed flow cytometry analysis on the peripheral blood of all mice, analyzing the frequency of T, natural killer (NK), Myeloid, and B cells (Figure S4) (Figures 5A–5F). There were no significant differences in the frequency of T, NK, and Myeloid cells across any conditions (Figures 5A–5D), indicating that the *Btk* editing did not skew blood cell lineage development. To ensure full coverage of all cells within the Myeloid compartment, both Ly6G and CD11b FACS markers were used.

In analyzing B cells, both CD19 and B220 flow cytometry markers were used to increase confidence in the analysis. The intron 1 and exon 2 editing conditions showed significantly higher frequencies of B cells, compared with the mock control mice, with no significant difference compared with the WT transplant conditions with both CD19 and B220 B cell markers (Figures 5E and 5F).

To fully confirm the maintenance of Btk lineage-specific expression of immune cells of edited mice, intracellular flow cytometry staining for the Btk protein was performed on peripheral blood immune cells in addition to immunophenotypic extracellular staining. There were no significant differences in the frequency of Btk+ immune cells across any of the conditions or cell types, giving increased confidence in the maintenance of Btk lineage-specific expression (Figure 5G). Additionally, the expression level, or mean fluorescence intensity across each lineage was not significantly different across any given condition (Figure S5), further indicating maintenance of lineage-specific Btk expression. Low levels of Btk expression were detected in T and NK cells, which is likely an artifact based on the sensitivity of the flow cytometry analysis. Independent of this, it is important to note that despite the detection of Btk^+^ T and NK cells, both the percentage positive and MFI are the same in our edited conditions as compared with controls (Figures 5G and S5)—validating the likely rationale of the limited sensitivity of flow cytometry detection.

XLA is characterized by the extreme paucity or complete absence of antibodies in patients due to the lack of mature B cells.^39^ An effective gene therapy strategy should not only allow for a reconstitution of antibody production, but also an increase in all classes of immunoglobulins for full protection. This is especially relevant as the main treatment for XLA is immunoglobulin replacement therapy (IgRT), with IgG making up a majority of the immunoglobulins in the product.^40^ Due to the lack of other immunoglobulin classes in IgRT, XLA patients are increasingly susceptible to sinopulmonary infections.^41^ To quantify the production of secreted antibody classes, ELISA analysis was performed to measure total IgA, IgG, IgM, and IgE from serum from peripheral blood of all mice. Across all four classes of immunoglobulins, *Btk*-edited intron 1 and exon 2 conditions showed significantly increased antibody production above the levels of the mock transplant control, with no significant difference compared with the WT transplant control (Figures 5H–5K).

Last, a major function of B cells and antibodies is production of antigen-specific antibodies in response to the large variety of potential foreign antigens individuals could be faced. An effective gene therapy would not only allow for the production of variety of antibody classes (Figures 5H–5K) but also allow for the ability to respond to specific antigens to mount a humoral response. There are two types of antigens that B cells could respond to: T-independent and T-dependent. Mounting a humoral response to a T-dependent antigen requires not only BCR recognition of a foreign antigen, but also T cell help through both cytokine production and CD40/CD40-L interaction.^42^ Based on this, we sought to determine if the mice transplanted with *Btk*-edited Lin- cells could mount primary and secondary humoral responses, as it would indicate not only the ability of the B cells to recognize foreign antigens, but also their ability to interact with T cells to mount a humoral immune response.

To test the ability to mount T-dependent antigen responses, we immunized the mice with 4-Hydroxy-3-nitrophenylacetyl Chicken-Gamma-Globulin (NP-CGG), a well characterized T-dependent antigen and measured the ability to produce both a primary and secondary humoral response by ELISA analysis of NP-IgG1 antibody levels. *Btk*-edited intron 1 and exon 2 mice showed significant increases in both low and high-affinity NP-IgG1 antibody production levels above that of the mock control mice, with no significant differences relative to the WT transplant control condition (Figures 5L and 5M) (Tables S3 and S4). Additionally, a hallmark of a successful humoral response is a larger secondary response to immunization relative to primary immunization.^43^ This trend is showcased clearly for both low- and high-affinity NP-IgG1 antibody response to immunization, giving increased confidence in the responsiveness of *Btk*-edited B cells in the intron 1 and exon 2 conditions. Collectively, we have showcased the XLA mice receiving *Btk*-corrected stem cells maintained endogenous leukocyte immunophenotypic patterns, showed increased peripheral B cell production, maintained lineage-specific Btk expression patterns, displayed increased antibody production of all classes, and had the ability to respond to T-dependent antigens with primary and secondary responses.

## Discussion

In this study, we have successfully demonstrated the effectiveness of a site-specific *Btk* gene correction strategy in a Btk/Tec^−/−^ mouse model of XLA, showing significant improvement in B cell development, function, and antibody production. These findings successfully support the clinical potential of *BTK* gene editing for the treatment of XLA patients.

One important facet of these findings that gives confidence to the potential success of this therapy is the selective advantage of BTK-replete cells in the spleen that has been previously characterized,^9^^,^^13^ and is further supported by our findings. Despite only having ∼10%–15% *Btk* correction in the bone marrow of the mice transplanted with *Btk*-edited cells, we were able to detect the inserted *BTK* gene in 35% of cells in the spleens of these mice. The splenic niche provides adequate space and biological conditions to allow for expansion of Btk-replete B cell progenitors, and this can be seen at 24 weeks post-transplant.^4^^,^^44^

Additionally, another major benefit of a *Btk* site-specific insertion strategy is the maintenance of lineage and developmental state-specific Btk expression. The specific increase in the frequency of B cells achieved, without alterations of other immune cell compartments, will be critical for the long-term safety and success of this approach. This contrasts with the other main gene therapy strategy commonly used with hematopoietic stem cells using lentiviral vector-mediated gene addition, which may result in unregulated expression of the gene of interest driven by a ubiquitous promoter at multiple ectopic insertion sites. While lentiviral vector-based therapies have been clinically successful for multiple indications where precise control of transgene expression is not needed (ADA SCID, XSCID, XCGD, LAD I),^45^ there is concern that BTK overexpression or ectopic expression could make a lentiviral gene addition strategy potentially unsafe. Aberrant BTK expression levels or lineages could significantly increase the risk for oncogenesis or autoimmunity in treated XLA patients. Thus, it is critical that a *BTK* gene editing strategy results in proper BTK expression through B-lymphoid development and function.^9^ The use of the targeted gene insertion strategy should maintain endogenous transcriptional regulation and preserve lineage-specific expression of the BTK protein, providing a clinically safe therapeutic option for the treatment of XLA.^46^

Although these results are very encouraging, there are still limitations to our study that will need to be addressed in further studies to confirm the safety and long-term viability of this strategy. Off-target analysis of Cas9 dsDNA breaks in edited human cells using the clinically relevant human BTK intron 1 and exon 2 gRNAs has already been conducted using GuideSeq NGS analysis.^16^ However, more thorough assessment of genotoxicity related to the editing strategy and integration outcomes at the target site will be required for translation of this project to the clinic. Although CRISPR-Cas9 and HDR-mediated gene addition have been proven effective and safe in preclinical studies,^47^^,^^48^^,^^49^ there are general concerns over how Cas9 double-stranded DNA breaks will generally be tolerated in a clinical context. Another important facet of the clinical-translatability of this study is to confirm that the resulting disease correction seen is attributable specifically to long-term HSCs and not long-lasting progenitors, likely through secondary transplants of HSCs from our primary recipients into secondary BTK/TEC^−/−^ recipients combined with confirmation of equal *Btk*-genomic editing across all lineages. Confirmation of long-term HSC editing will prove critical to XLA patients displaying sustained B cell restoration and antibody production. This underlies the importance of improving delivery strategies and reducing toxicity, enhancing stem cell functionality, and increasing overall engraftment efficiency.^21^^,^^50^ While these studies could have been conducted in the murine XLA model, the authors found it more clinically relevant to conduct these studies in the cell product that will be used clinically—human XLA patient CD34^+^ hematopoietic stem and progenitor cells (HSPCs), of which more in-depth genomic and cellular outcomes of editing will be the subject of a subsequent manuscript.

Extensive characterization of Cas9 RNP-mediated effects genome-wide will be required in subsequent experiments for human CD34+ HSPCs to confirm the safety of editing reagents, with a particular emphasis on off-target activity. With increased development of new strategies to analyze off-target editing,^51^ each with strengths and limitations, a combination of various strategies would likely be the ideal approach to increase confidence in the safety of this site-specific correction strategy. Strategies for assessing off-target editing *ex vivo* should then be followed with long-term assessment of hematopoietic reconstitution in additional preclinical models.^52^ However, because XLA is caused by a BTK null phenotype, any perturbations within the coding region of *BTK* (insertions/deletions at the editing site) will likely not result in novel deleterious effects, highlighting a key advantage to gene editing for complete loss-of-function disorders.^53^

Another major factor affecting the health and potential of transplanted cells is the effect of rAAV6-mediated homologous donor transfer of the therapeutic *BTK* gene. Our findings highlighted that the viability of Lin- cells decreased following electroporation and AAV transduction as compared with our non-edited mock control, which could directly negatively affect downstream engraftment, particularly of true long-term stem cells. We have previously shown that various commercial manufacturers of research-grade AAV have significantly different “purity” levels of rAAV genomes within their preps, and that these differences can have major impacts of stem cell viability, genome editing, clonogenic potential, and transcriptional response following transduction.^21^ Selection of a high-quality AAV prep that can maximize HDR editing while minimizing the deleterious effects of AAV transduction on stem cell potential will be critical for an effective site-specific correction gene therapy strategy.

In statistical analyses of the effects of *Btk* intron 1 and exon 2 edited cells, these conditions were compared with the mock transplant group (unedited Btk/Tec^−/−^ male donors into Btk/Tec^−/−^ female recipients) as a negative control, and WT transplant group (C57bl6/J donors into Btk/Tec^−/−^ recipients) as a positive control. Use of control groups that had undergone conditioning and transplant accounts for these experimental variables, which may result in incomplete immune reconstitution in patients who receive similar conditioning and stem cell transplantation.^54^^,^^55^^,^^56^

Because the low number of *Btk*-corrected cells in the bone marrow resulted in major increases in mature B cells in the spleen, low levels of engraftment may be beneficial for XLA patients. This opens the possibility of using a reduced-intensity conditioning (RIC) regimen for gene therapy of XLA patients receiving transplantation of *BTK*-corrected HSPCs, which would have lower transplant-related complications than fully myeloablative conditioning.^57^ However, gene correction of sufficient numbers of stem cells will be needed to allow consideration of RIC conditioning.

For all studies, we performed head-to-head comparisons of the intron 1 and exon 2 *Btk* gene targeting strategies. This was based on previous *in vitro* studies within human immortalized cell lines and primary human HSPCs that showed achievable high rates of editing at both *BTK* sites.^16^ Both the intron 1 and exon 2 target sites allow for integration at or near the very beginning of the coding region for the BTK protein, allowing either target to be an effective treatment for nearly all patients with *BTK* mutations, independent of their location in the *BTK* gene. One critical consideration is that the intron 1 target site requires the addition of a splice-acceptor site in the *BTK* donor to get proper splicing of intron 1 to form a functional *BTK* cDNA after homologous recombination. Should the intron 1 target site move forward in preclinical studies, special emphasis will be required to ensure proper splicing of intron 1 of the donor transgene is occurring on the RNA level to ensure proper *BTK* mRNA and protein production.

This study confirms the validity of the site-specific *Btk* editing approach for the treatment of XLA. Even though this study focused on gene correction for the treatment of XLA, it provides further meaningful evidence that a site-specific editing approach is a true possibility for the treatment of many inborn errors of immunity. Although there are still major challenges that remain between this murine model study and clinical translation, such as the optimization of editing conditions, confirmation of minimal genotoxicity, and feasibility of performing these studies at larger scales, our data indicate that this strategy holds promise for the clinical treatment of XLA patients. Together, these studies represent the application of a mutation-independent, one-time, site-specific *BTK* gene insertion strategy capable of giving XLA patients the possibility of functional B cell reconstitution without the need for lifelong immunoglobulin replacement therapy.

## Materials and methods

### 32D cell culture

The 32D cell line was obtained from ATCC (Manassas, VA). Cells were maintained in R-10 (RPMI [Corning Life Sciences, Corning, NY] + 10% FBS [Omega Scientific, Tarzana, CA] + 1× penicillin/streptomycin/glutamine [Fisher Scientific, Waltham, MA]) supplemented with 10 ng/mL murine-IL3 (Thermo Fisher Scientific, Waltham, MA) at 37°C with 5% CO_2_ and were confirmed to be negative for mycoplasma by testing with MycoAlert (Lonza Biologics).

### Generation of Btk^−/−^ 32D cell line

32D cells were modified to knockout the *Btk* gene by electroporation of SpCas9 recombinant protein (QB3 Macrolab, UC Berkeley, Berkeley, CA) complexed to single guide RNA (sgRNA) targeting exon 12 of the *Btk* gene (5′-GCGTGGAACACACAACGTAATGG-3′) (Synthego, Redwood City, CA) R-20: (RPMI [GIBCO] + 20% FBS [GIBCO] + 1× penicillin/streptomycin/glutamine [Gemini Bio Products, Sacramento, CA]) supplemented with 10 ng/mL murine-IL3 (Peprotech, Cranbury, NJ). Primers for amplification of the *Btk* locus to confirm knockout were as follows:

BTK-E12F: 5′- CGTGTTATGGTTCCAGTGATATGG -3′;

BTK-E12R: 5′-ATTGAAGGGATGGGGAGATGTGTG -3′.

PCR-amplified exon 12 genomic DNA from single clones was analyzed via Synthego Inference of CRISPR Edits (ICE) analysis (Synthego, Redwood City, CA) to confirm complete DNA knockout. Absence of Btk protein in cell lines was confirmed by western blot analysis of Btk protein (see western blot protocol for further information). Cells were subsequently cultured in R10 + mIL3 at 37°C with 5% CO_2_.

### The Btk/Tec^−/−^ mice

Btk^−/−^ mice (B6:129S-Btk^tm1wk^/J – stock # 002536) were obtained from The Jackson Laboratories. The Tec^−/−^ mice were a generous gift of Wilfred Ellmeir, PhD, from the University of Vienna, Austria.^22^ The two strains were crossed until mice homozygous for both the Btk and Tec mutations were obtained and then the Btk/Tec^−/−^ mice were maintained as a colony under conditions for immune-deficient mice. All mice handling and experimental protocols were maintained under the approved oversight of institutional review board.

### Lin- cell harvest/culture

Femurs and tibias from WT Bl6 and Btk/Tec^−/−^ mice were crushed using mortar and pestle to harvest total bone marrow cells. Cells were then passed through a 70-μM filter (Corning Life Sciences, Tewksbury, MA). Following 70 μM filtration, total bone marrow cells underwent Lin- cell purification via Direct Lineage cell depletion kit (Miltenyi Biotec; Bergisch Gladbach) following the manufacturer’s protocol, including an additional 30 μM filtration step (Miltenyi Biotec; Bergisch Gladbach). Negatively selected Lin- cells then underwent counting via hemacytometer and trypan blue exclusion followed by plating at 5 × 10^5^ cells/mL in Stemspan SFEMII medium (Stem Cell Technologies, Vancouver, BC, Canada) containing mIL3 (20 ng/mL), hFLT3-L (100 ng/mL), mSCF (100 ng/mL), and hIL11 (100 ng/mL) (all from Thermo Fisher Scientific, Waltham, MA) for 48-h prestimulation. Following editing protocols, Lin- cells were then plated and maintained at 5 × 10^5^ to 1 × 10^6^ cells/mL for the duration of experimentation.

### 32D *Btk* editing

32D cells (ATCC CRL-11346) were electroporated at 85% confluency. Cells were counted on a ViCell (Beckman Coulter, Brea, CA) and 5 × 10^5^ cells per condition were centrifuged at 90 × *g* for 15 min at room temperature (RT), resuspended in 20 μL of SF electroporation buffer (Lonza, Basel, Switzerland). During the 15-min spin, 140 pmol sgRNA targeting either intron 1 (sgRNA-intron1: 5′- GGGCTGAGGCAGAACCAGGT-3′) or exon 2 (sgRNA-exon2: 5′- TGTGTACAGTCAAGAGAAAC-3′) were combined with 100 pmol of rCas9 nuclease protein for 15 min at RT for RNP complex formation. Twenty microliters of resuspended cells were then combined with the preformed RNP complexes. Cells were electroporated using the CV-137 setting on the Amaxa 4D Nucleofector X Unit (Lonza). Immediately after electroporation, to measure allelic disruption, the RNP-only condition cells were rested in 16-well electroporation strips (Lonza) for 10 min at RT and then recovered with 480 μL of R-10 medium.

Cells that received the rAAV-delivered *BTK* homologous donor were also rested for 10 min then recovered in 100 μL R-10 medium and added to sterile flow cytometry tubes containing the rAAV6 vector with the *BTK* cDNA and flanking homology arms (Virovek Inc, Houston, TX) at desired MOIs diluted up to 50 μL with PBS. Electroporated cells + rAAV6 (total 150 μL) were then incubated at 37°C with 5% CO_2_ for 2 hours, with gentle vortexing every 20 min. After 2 h, 350 μL R-10 + mIL3 were added to flow tubes and the entire contents (500 μL) were transferred to 24,504-well plates. Eighteen to 24 h post-transduction, cells were harvested and counted using hemacytometers and trypan blue exclusion calculation of viability. Following counting, cells were centrifuged at 300 × *g* for 8 min and resuspended in 500 μL fresh R-10 medium.

### Lin- cell editing

Lin- cells were electroporated at 85% confluency. Cells were counted via hemacytometer and trypan blue exclusion and cells were centrifuged at 90 × *g* for 15 min at RT and resuspended in 100 μL of Maxcyte Cytiva electroporation buffer (Maxcyte; Rockville MD). Due to the high cell number capacity capable of being electroporated using the Maxcyte system, the number of cells electroporated varied from 5 × 10^5^ up to 8 × 10^6^ total cells depending on the experiment, with no effect on Cas9-mediated allelic disruption (data not shown). During the 15-min spin, 280 pmol sgRNA targeting either intron 1 (sgRNA-intron1: 5′- GGGCTGAGGCAGAACCAGGT-3′) or exon 2 (sgRNA-exon2: 5′- TGTGTACAGTCAAGAGAAAC-3′) were combined with 200 pmol of rCas9 nuclease protein for 15 min at RT for RNP complex formation. One hundred microliters of resuspended cells were then combined with the preformed RNP complexes. Cells were electroporated in the OC-100 cuvette using the optimization 4 setting on the Maxcyte ATX Unit (Maxcyte, Rockville, MD). Immediately after electroporation, to measure allelic disruption, the RNP only condition cells were rested in OC-100 electroporation cuvette (Maxcyte, Rockville, MD) for 10 min at RT and then recovered with 480 μL of SFEMII medium containing cytokines listed in Lin- cell culture section.

Cells that received the *BTK* homologous donor via rAAV transduction were electroporated then recovered in 150 μL SFEMII medium with cytokines and added to sterile flow cytometry tubes containing rAAV6 vector (Virovek Inc, Houston, TX) at desired MOIs diluted up to 50 μL with PBS. Electroporated cells + rAAV6 (total 200 μL) were then incubated at 37°C with 5% CO_2_ for 2 hours, with gentle vortexing every 20 min. After 2 h, 300 μL SFEMII + cytokines was added to flow tubes and the entire contents (500 μL) were transferred to 24-well plates. Eighteen to 24 h post-transduction, cells were harvested and counted using hemacytometers and trypan blue exclusion calculation of viability. Following counting, cells were centrifuged at 300 × *g* for 8 min and resuspended in 500 μL fresh SFEMII medium.

### Analysis of allelic disruption

Five days post electroporation/transduction, editing outcomes were measured from genomic DNA (gDNA) extracted using PureLink Genomic DNA Mini Kit following the manufacturer’s instructions (Invitrogen/Thermo Fisher Scientific, Waltham, MA). To measure allelic disruption, genomic DNA extracted from cells was PCR amplified. Due to the proximity of each gRNA site, the same amplification primers were used for both intron 1 and exon 2 target sites:

mBTK-Intron1F: 5′- GGGACTGTGGAAGAAGGAGC-3′

mBTK-Intron1R: 5′- GGCAGCCTAAAAGAACCTGG-3′

PCR-amplified products were then sent for Sanger sequencing using the mBTK-Intron1F primer and allelic disruption quantified using ICE analysis (Synthego, Redwood City, CA).

### ddPCR for analysis of site-specific *Btk* integration

ddPCR analysis was conducted as in Long et al.^58^ A primer set was specifically designed to amplify site-specific *Btk* insertion with one primer binding just upstream of the integration site in the endogenous *Btk* locus, and another primer binding within the cDNA sequence at an exon-exon junction. This way, amplification will only occur if the *BTK* cDNA integrates specifically at its endogenous locus. The quantification of these integration events is the normalized to a reference housekeeping gene.

Reaction mixtures of 20-μL volume were prepared containing 1x ddPCR Master Mix (Bio-Rad, Hercules, CA), relevant primers and probe (400 nM primers and 100 nM probe), 20 to 100 ng of input gDNA, 0.05× EcoRV restriction enzyme (New England Biolabs, Ipswich, MA), with the remaining volume supplemented with DNAse-free water and incubated for 1 h at 37°C. Droplet generation was performed as described in Hindson et al.^59^ and the droplet emulsion was transferred to a 96-well propylene plate (Eppendorf, Hamburg, Germany) and amplified in a conventional thermal cycler (T100 thermal cycler, Bio-Rad, Hercules, CA). Thermal cycling conditions consisted of 95°C for 10 min, 94°C for 30 s, 60°C for 1 min, and 72°C for 2 min (44 cycles); 72°C for 10 min (1 cycle); and 12C hold. After PCR, the 96-well plate was transferred to a droplet reader (Bio-Rad, Hercules, CA). Acquisition and analysis of the ddPCR data were performed with the QuantaSoft software (Bio-Rad, Hercules, CA), provided with the droplet reader. The integration event frequency was calculated by dividing the concentration (copies per microliter) of successful *BTK* integration events by the concentration of the RPP30 reference gene, used to normalize per murine genome. The primers and probes are listed below:

RPP30 F: tgtctgaacagaagtgcctg

RPP30 R: tgggagacattacccattcc

RPP30 probe: acagtgagtcccccttgcatctgctggt

mBTK ddPCR F: CACCTGGATGGTAGTCACTGTGTATT

mBTK ddPCR R: ATCTTTTCCACGTCGATGCT

mBTK probe: TCGAAGTCGTACTCGTAG.

### ddPCR analysis of Y Chromosome donor cell engraftment

ddPCR analysis was conducted as in Long et al.^58^ To quantify engraftment, a Y chromosome-specific DNA is normalized to a reference housekeeping gene. Reaction mixtures of 20 μL volume were prepared containing 1x ddPCR Master Mix (Bio-Rad, Hercules, CA), relevant UC378 primers and probe (400 nM primers and 100 nM probe), 1× Uty primer probe (Thermo Fisher 4400291, Waltham, MA), 20 to 100 ng of input gDNA, 0.05× DraI restriction enzyme (New England Biolabs, Ipswich, MA), with the remaining volume supplemented with DNAse-free water and incubated for 1 h at 37°C. Droplet generation was performed as described in Hindson et al^59^ and the droplet emulsion was transferred to a 96-well propylene plate (Eppendorf, Hamburg, Germany) and amplified in a conventional thermal cycler (T100 thermal cycler, Bio-Rad, Hercules, CA). Thermal cycling conditions consisted of 95°C for 10 min; 94°C for 30s, 60°C for 1 min (54 cycles); 98°C for 10 min (1 cycle); and 12C hold. After PCR, the 96-well plate was transferred to a droplet reader (Bio-Rad, Hercules, CA). Acquisition and analysis of the ddPCR data were performed with the QuantaSoft software (Bio-Rad, Hercules, CA), provided with the droplet reader. The engraftment frequency was calculated by multiplying the concentration (copies per microliter) of Y chromosome DNA ×2 (to account for one Y chromosome copy/genome) by the concentration of the UC378 reference gene (with two copies), used to normalize per murine genome. The primers and probes are listed below:

**uc378-ddPCR F**: CGCCCCCTCCTCACCATTAT

**uc378-ddPCR R**: CATCACAACCATCGCTGCCT

**uc378 probe (HEX):** TTACCTTGCTTGTCGGACCAAGGCA.

### Western blot analysis

Western blotting was performed as in Gray et al.^16^ Cell pellets were washed once with PBS then lysed in RIPA cell extraction buffer (Thermo Fisher Scientific, Waltham, MA) with added HALT protease inhibitor (Thermo Fisher Scientific, Waltham, MA) at a 1× concentration for 45 min on ice with vigorous vortexing every 15 min. Samples were then spun at 17,000 × *g* for 20 min at 4°C before transferring the supernatant to a fresh tube. Lysate concentrations were determined using the Pierce BCA protein assay (Thermo Fisher Scientific, Waltham, MA) following the manufacturer’s protocol. Lysates were then combined in a mixture with 40 μg total protein with 1× NuPAGE LDS Sample Buffer (Thermo Fisher Scientific, Waltham, MA), 1× NuPAGE Sample Reducing Agent (Thermo Fisher Scientific, Waltham, MA), and supplemented up to the appropriate total volume with RIPA lysis buffer. WT 32D lysates were diluted to contain equivalent amounts of integrated *BTK* gene copies according to integration rates detected via ddPCR for Intron1 and exon 2 conditions, using lysate from the Btk-deficient 32D cells to keep constant the total amount of protein loaded per lane to allow for valid loading controls.

Samples were then heated to 95°C for 10 min before loading into a 4%–12% Bis-Tris gel (Thermo Fisher Scientific, Waltham, MA). Gels were then run at 30V for 20 min followed by 140V for 1 h, then transferred onto 0.45-μM Nitrocellulose membranes according to standard wet transfer protocols (Thermo Fisher Scientific, Waltham, MA). The membrane was blocked with 5% milk in 1× Pierce TBS Tween 20 buffer (Thermo Fisher Scientific, Waltham, MA) for 1 h. Membranes were split into discrete portions to probe for either Btk or Beta-Actin proteins and incubated in a 1:1,000 dilution of primary antibody for BTK (Cell Signaling Technology, Danvers, MA) or Beta-actin (Cell Signaling Technology, Danvers, MA), shaken overnight at 4°C. Membranes were washed three times for 10 min with 1× TBS Tween and incubated in 5% milk with horseradish peroxidase (HRP)-conjugated, species-specific secondary antibodies for 90 min at RT (BD Biosciences, San Diego, CA). Secondary antibodies were then washed three times for 10 min with 1× TBS Tween. Blots stained with HRP-conjugated antibodies were treated with Pierce ECL Plus Western Blotting Substrate (Thermo Fisher Scientific, Waltham, MA). All blots were imaged via Typhoon Imaging System (Marlborough, MA) under the enhanced chemiluminescence (ECL) setting.

### Btk/Tec^−/−^ mouse transplant/radiation

Animals were handled in laminar flow hoods and housed in a pathogen-free colony in a biocontainment vivarium. Young adult (4–6 weeks old) Btk/Tec^−/−^ female recipients were irradiated at a dose of 450 rads with a cesium-137 source twice: 24 h before and immediately before transplantation. Twenty-four hours post editing, Lin- cells were counted, washed, and resuspended at ∼1 × 10^7^ cells/mL in sterile PBS and 1 × 10^6^ total cells loaded into syringes. Recipient mice were then injected with ∼1 × 10^6^ untreated Btk/Tec^−/−^, *Btk* intron 1 edit, *Btk* exon 2 edited, or WT C57/Bl6 Lin- cells/mouse via retro-orbital injection and allowed to engraft over 24 weeks. After 24 weeks, mice were euthanized by CO_2_ inhalation followed by cervical dislocation. Bone marrow, peripheral blood, and spleens were harvested for endpoint analysis.

### Immunization with NP-CGG

At 19 weeks post-transplant, mice were given 100 mg of NP(40)-CGG (Biosearch Technologies, Novato, CA) in 100 μL of Imject alum, which serves as an adjuvant (Thermo Fisher Scientific, Waltham, MA) followed by a secondary immunization with the same reagents 28 days post primary immunization. Serum was collected the day prior to primary immunization, 10 days post primary immunization, the day prior to secondary immunization, and 10 days post-secondary immunization, which also served as the day of full tissue harvest.

### Flow cytometry for blood, spleen, and bone marrow

Flow cytometry was performed on single-cell suspensions from peripheral blood, spleen, and bone marrow cells resuspended in PBS +1% BSA. All extracellular flow stains were done according to manufacturer protocols with 30-min RT incubation in the dark. All samples received a 10-min anti-CD16/32 Fc Block (BD Biosciences, Milpitas, CA) prior to staining with extracellular antibodies. Peripheral blood samples underwent lysis using 1× RBC lysis buffer (Thermo Fisher) according to manufacturer protocols prior to extracellular staining. For Btk intracellular flow cytometry, peripheral blood samples underwent extracellular staining followed by fixation and permeabilization using Cytofix/Cytoperm kit (BD Biosciences, Milpitas, CA) according to the manufacturer’s protocol followed by intracellular Btk protein staining, subsequent washes, and flow cytometry analysis. All flow cytometry recordings were conducted using BD LSRII cytometer (BD Biosciences, Milpitas, CA) and analysis conducted using FlowJo flow cytometry analysis software. To see flow antibodies used for panels please see the reagents Table S5.

### Next-generation sequencing for B cell receptor diversity

Library preparation was performed using the NEBNext Mouse Immune Sequencing kit (New England Biolabs, Ipswich, MA) according to the manufacturer’s instructions with 100 ng of input RNA isolated from single-cell isolates from splenic tissue. RNA extraction was conducted using the Qiagen RNeasy Mini Kit (Qiagen Sciences, Germantown, MD). Next-generation sequencing (NGS) was performed using MiSeq V3 600 cycles, with 2 × 300 paired end sequencing, for a total of 25M reads, with 10% PhiX DNA spiked in and a final pooled library concentration of 8 pM. Upon completion of NGS, fastq files were run through the REpertoire Sequencing TOolkit (pRESTO) NEBNext Immune Sequencing Kit workflow (https://usegalaxy.org/published/workflow?id=51f936d1a4e849fa) (New England Biolabs, Ipswich, MA) to generate .tsv files containing all unique VDJ recombinant sequences and frequencies of these sequences. The .tsv files were then run through a custom script on R-studio to generate resultant pie charts displaying diversity as well as calculations of Shannon diversity index scores.

### ELISA for total immunoglobulins and NP-specific IgG1 antibodies

Serum was collected by allowing peripheral blood to clot in Eppendorf tubes for 1–2 h at RT, spinning samples in a centrifuge at 3,000 × *g* for 10 min at 4°C. The uppermost layer not containing the clot was then isolated, transferred to a new Eppendorf tube, and frozen at −80°C until ELISA analysis.

ELISA analysis was conducted as in Lalani et al.^60^ For total Ig ELISA (IgG, IgM, IgE, IgA), 96-well Nunc Maxisorp plates (Thermo Fisher Sciences, Waltham, MA) were coated with 100 μL of 10 μg/mL of isotype-specific anti-mouse capture antibody diluted in PBS and incubated overnight at 4°C. Plates were then washed 2× with 200 μL/well of wash buffer (PBS + 0.05% tween 20) followed by addition of 200 μL blocking buffer (PBS + 2.5% BSA) (Sigma-Aldrich, St. Louis, MO) for 1 h at RT. During blocking, serum samples were diluted in blocking buffer accordingly: IgG and IgM: 1:1 × 10^5^, 1:5 × 10^5^, 1:1 × 10^6^, 1:1 × 10^7^; IgA: 1:100, 1:1,000, 1:1 × 10^4^; IgE: 1:25, 1:125, 1:625. With Ig isotype standards (Southern Biotech, Birmingham, AL) diluted in blocking buffer starting at 500 ng/mL followed by eight 2-fold serial dilutions. After blocking, plates were washed three times with 200 μL wash buffer followed by addition of 100 μL of the Ig Isotype standards and diluted serum samples to appropriate wells and overnight incubation at 4°C. The next day, plates were washed 3× with 200 μL of wash buffer followed by addition of 100 μL of isotype-specific HRP-conjugated anti-mouse antibody and a 2-h incubation at RT. Plates were then subsequently washed five times with 200 μL of wash buffer followed by addition of 100 μL of RT TMB substrate solution (Thermo Fisher). After short incubation, 1N H_2_SO_4_ was added to stop the reaction. Plates were then read out using a photospectrometer (Teacan sciences, Chapel Hill, NC), measuring absorbance values at a wavelength of 450 nm.

For NP-IgG ELISA, Nunc Maxisorp plates were coated with 100 μL of NP(4)-BSA (high-affinity) or NP(40)-BSA (Southern Biotech, Birmingham, AL) (low-affinity) at 10 μg/mL overnight at 4°C. The next day, plates were washed and blocked as in the above total Ig ELISA protocol. During blocking, serum samples were diluted at 1:500, 1:2,500, and 1:12,500 and the Np-IgG1 standard control antibody (Abcam, Burlingame, CA) diluted starting at 250 ng/mL followed by eight 2-fold serial dilutions. Plates were then washed three times with 200 μL of wash buffer, and 100 μL of diluted serum and standards were added to each well, and plates were incubated at 4°C overnight. The next day, plates were washed as above and incubated with 10 μg/mL of HRP-conjugated anti-mouse IgG1 (Southern Biotech, Birmingham, AL) antibody for 2 h at RT. All subsequent washes, incubations, and detections were performed as detailed above.

### Quantification and statistical analysis

In all figures, *n* represents independent biological replicates and data are represented as mean ± standard deviation (SD). Statistical analysis was performed using GraphPad Prism software and *p* values were calculated from the two-tailed unpaired t test or multiple t test, unless otherwise noted in the figure legend: ∗*p* < 0.05; ∗∗*p* < 0.01, ∗∗∗*p* < 0.001, and ∗∗∗∗*p* < 0.0001.

## Data availability

The data that support the findings of this study are available from the corresponding author upon reasonable request.

## Acknowledgments

These studies were supported by grants from the California Institute for Regenerative Medicine (DISC2- DISC2-12111, D.B.K.), the Immune Deficiency Foundation (2023 IDF Michael Blaese Research Grant Award, D.B.K.), 10.13039/100000209The National Academy of Sciences Ford Predoctoral Fellowship (C.L.); the UCLA Molecular Biology Interdepartmental Program Whitcome Fellowship (C.L.); and the UCLA Division of Graduate Education Dissertation Year Award (C.L.). We thank Dr. Wilfred Ellmeir, PhD from the University of Austria for providing breeding pairs of the Tec^−/−^ mice. We thank the Flow Cytometry Core and the DNA Sequencing Core of the Eli & Edythe Broad Center of Regenerative Medicine and Stem Cell Research provided essential support. We thank the UCLA Behavioral Testing Core (BTC) for conducting all behavioral studies. We thank the lab of Dr. Kenneth Dorshkind, specifically Encaron Monetcino for their expertise in B cell flow-cytometry antibody panel design.

## Author contributions

D.B.K. conceived of the project, supervised the research studies, and co-wrote the manuscript. C.L. led all of the laboratory studies and was the primary author of the manuscript. A.M., A.C., S.C., J.D.L., and J.M.S. performed laboratory studies. X.W. performed all of the *in vivo* mouse work. Z.R provided technical advice.

## Declaration of interests

The authors have no financial interests in the work described here.

## Supplemental information

Document S1. Figures S1–S5 and Tables S1–S4

Table S5. Reagents

Document S2. Article plus supplemental information
