# Supplementary material for: Hematopoietic stem cell gene therapy for the treatment of X-linked agammaglobulinemia
Source: Mol Ther Methods Clin Dev. 2025 Aug 12;33(3):101555. doi: 10.1016/j.omtm.2025.101555 (PMC12410350; doi:10.1016/j.omtm.2025.101555)
Supplement: Document S1. Figures S1–S5 and Tables S1–S4 [file mmc1.pdf]

OMTM, Volume *33*

Supplemental information

Hematopoietic stem cell gene therapy

for the treatment of X-linked agammaglobulinemia

Christopher R. Luthers, Annika Mittelhauser, Aurelien Colamartino, Xiaomeng Wu, Samuel Cirigliano, Joseph D. Long, Julie M. Sanchez, Zulema Romero, and Donald B. Kohn

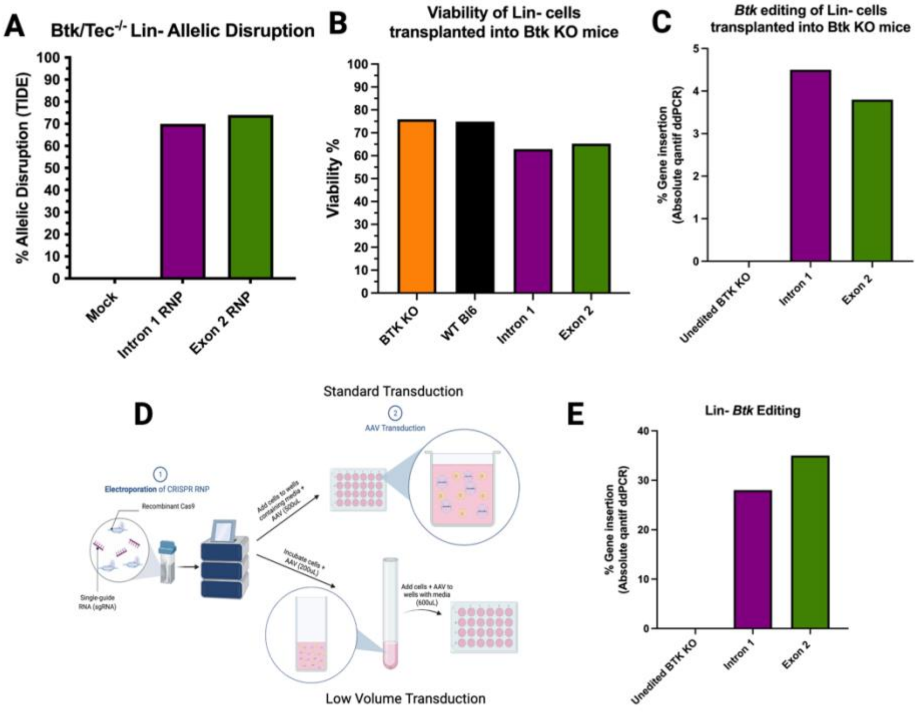

Figure S1: Alterations to Transduction Protocol Significantly Improves *Btk* Editing Efficiency. Lin- cells were isolated from freshly harvested bone marrow cells and edited in figure 1 before transplanting into Btk/Tec$^{-/-}$ mice. **(A)** Genomic DNA was isolated from Lin- cells which only received the Cas9 RNP followed by PCR amplification and Indel analysis using Synthego ICE software. **(B)** Lin- cells were counted using hemacytometer and trypan blue exclusion to determine viability. **(C)** Genomic DNA was harvested from Intron 1 and Exon 2 edited Lin- cells followed by in-out ddPCR analysis of Site-specific *BTK* cDNA insertion. **(D)** Following low editing efficiencies in Lin- cells, a new "Low Volume" transduction method was adopted in which followed electroporation, cells were incubated in 150-200uL total volume of cells and rAAV6 virus containing the *BTK* transgene for 2 hours with gentle vortexing, followed by transfer to a larger 24 well plate with more media. **(E)** Following alterations to editing protocols, genomic DNA was harvested from edited Lin- cells followed by in-out ddPCR analysis of site-specific *BTK* cDNA insertion, showing improved editing efficiencies.

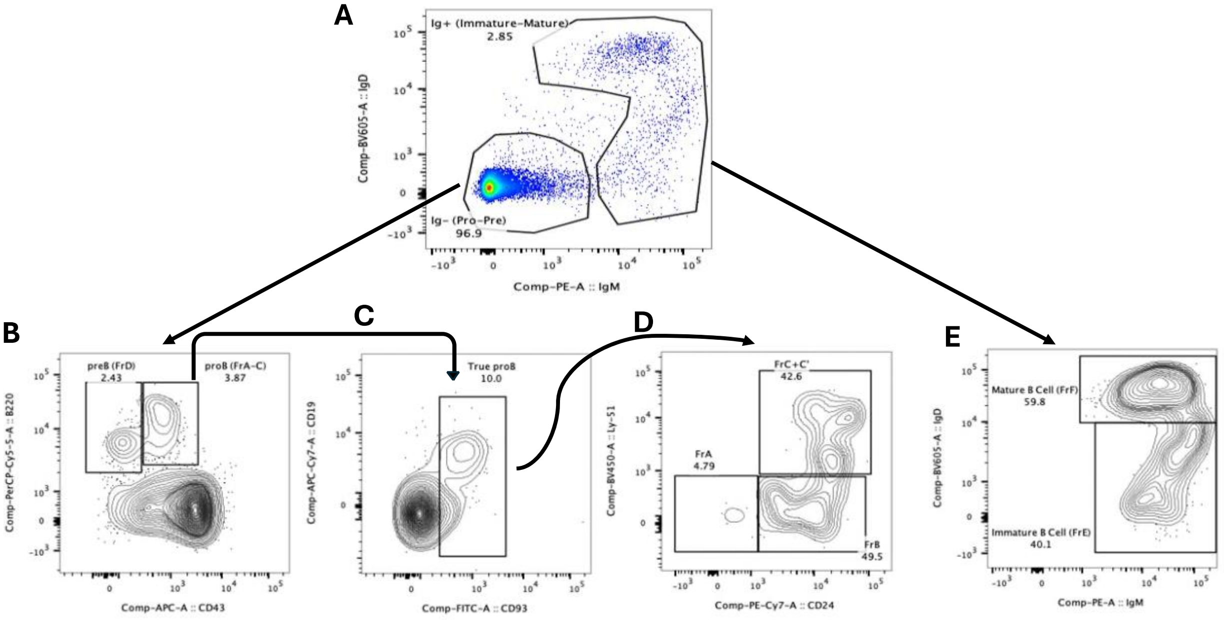

Figure S2: flow cytometry Staining Workflow for Bone Marrow B-Progenitors. Single cell-suspended total bone marrow cells underwent flow cytometry analysis. First, cells were gated for lymphocytes and single cells using FCS and SSC staining, followed by gating of live cells using a live cell exclusion stain (not shown). **(A)** Cells were then gated for Immunoglobulin- and Immunoglobulin+ using IgM and IgD markers to distinguish between Pre/Pro and Immature/Mature B cells. **(B)** Ig- cells were then stained with B220 and CD43 to distinguish between Pro (B220^{+}, CD43^{Lo}) and PreB (B220^{+}, CD43^{HI}) cells. **(C-D)** Pro B-cells were then further sub-sectioned into smaller fractions using CD93, CD19, Ly51, and CD24 Markers. **(E)** Ig+ cells were then further gated using IgM and IgD markers to distinguish between immature (IgM^{+}, IgD^{-}) and mature (IgM^{+}, IgD^{+}) B cells. All gates were drawn using single stain, fluorescence minus one (FMO), and antibody isotype controls for staining.

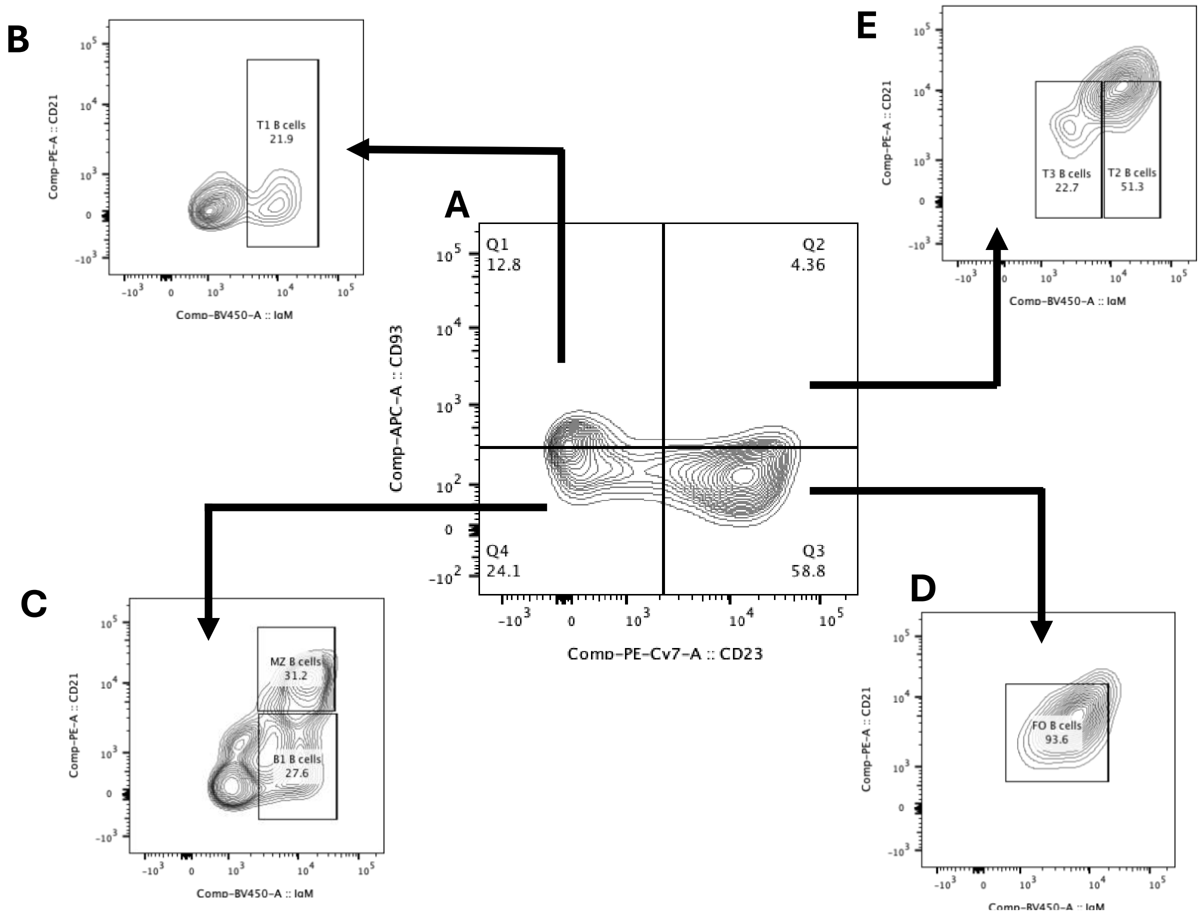

Figure S3: flow cytometry Staining Workflow for Immunophenotyping of Peripheral Blood Immune cells. Total Peripheral Blood cells underwent red blood cell lysis followed by flow cytometry analysis. First, cells were gated for lymphocytes and single cells using FCS and SSC staining, followed by gating of live cells using a live cell exclusion stain and total leukocytes using a CD45 marker (not shown). Cells were then further gated to distinguish between **(A)** CD19+ B-cells, **(B)** B220+ B-cells, **(C)** CD3+ T-cells, **(D)** NK1.1+ NK-cells, **(E)** Ly6G/C+ Myeloid-cells, and **(F)** CD11b+ Myeloid-cells. The experimental condition listed above represents all given plots in that column. All gates were drawn using single stain, fluorescence minus one (FMO), and antibody isotype controls for staining.

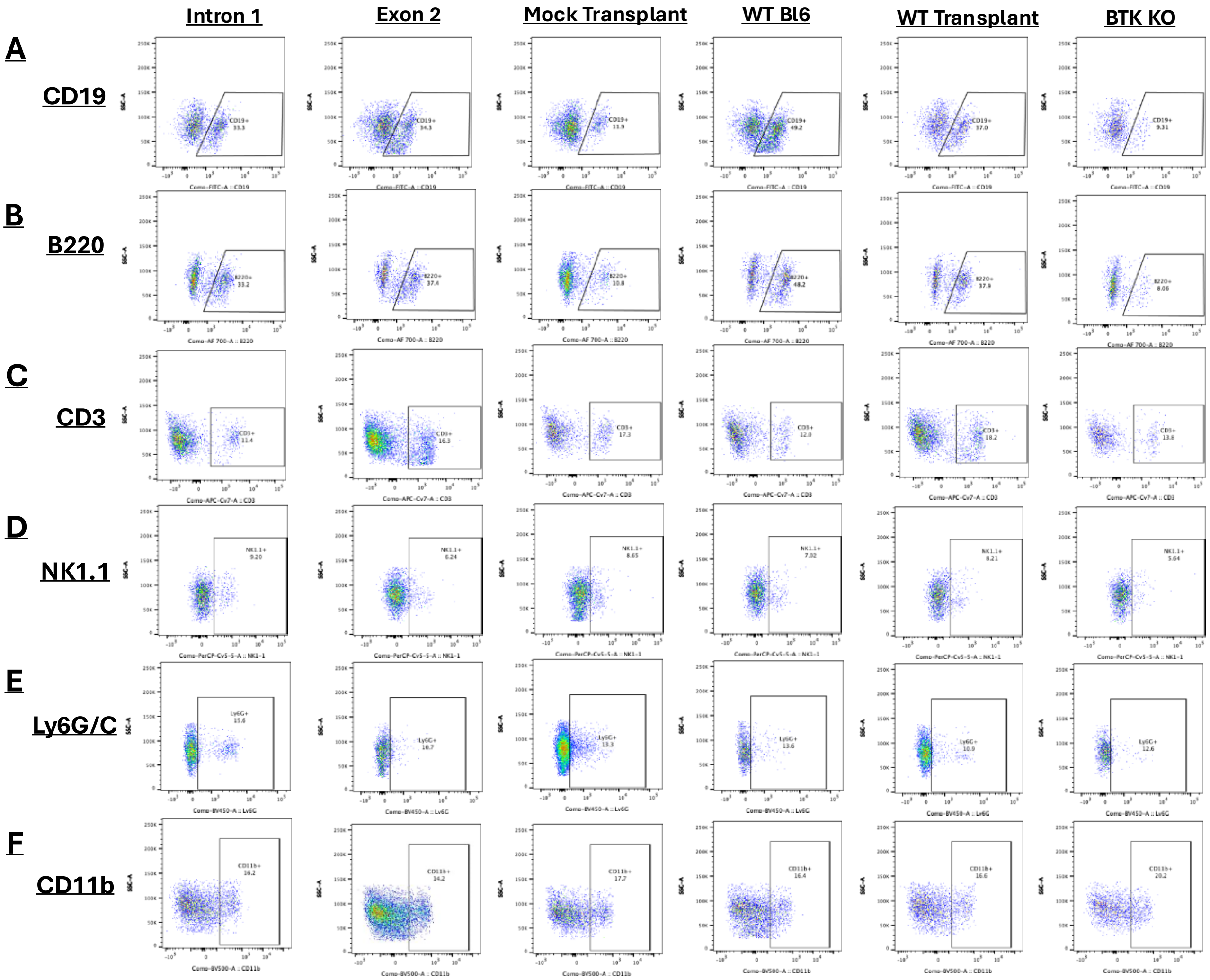

Figure S4: Flow cytometry Staining Workflow for Immunophenotyping of Peripheral Blood Immune cells. Total Peripheral Blood cells underwent red blood cell lysis followed by flow cytometry analysis. First, cells were gated for lymphocytes and single cells using FCS and SSC staining, followed by gating of live cells using a live cell exclusion stain and total leukocytes using a CD45 marker (not shown). Cells were then further gated to distinguish between **(A)** CD19+ B-cells, **(B)** B220+ B-cells, **(C)** CD3+ T-cells, **(D)** NK1.1+ NK-cells, **(E)** Ly6G/C+ Myeloid-cells, and **(F)** CD11b+ Myeloid-cells. The experimental condition listed above represents all given plots in that column. All gates were drawn using single stain, fluorescence minus one (FMO), and antibody isotype controls for staining.

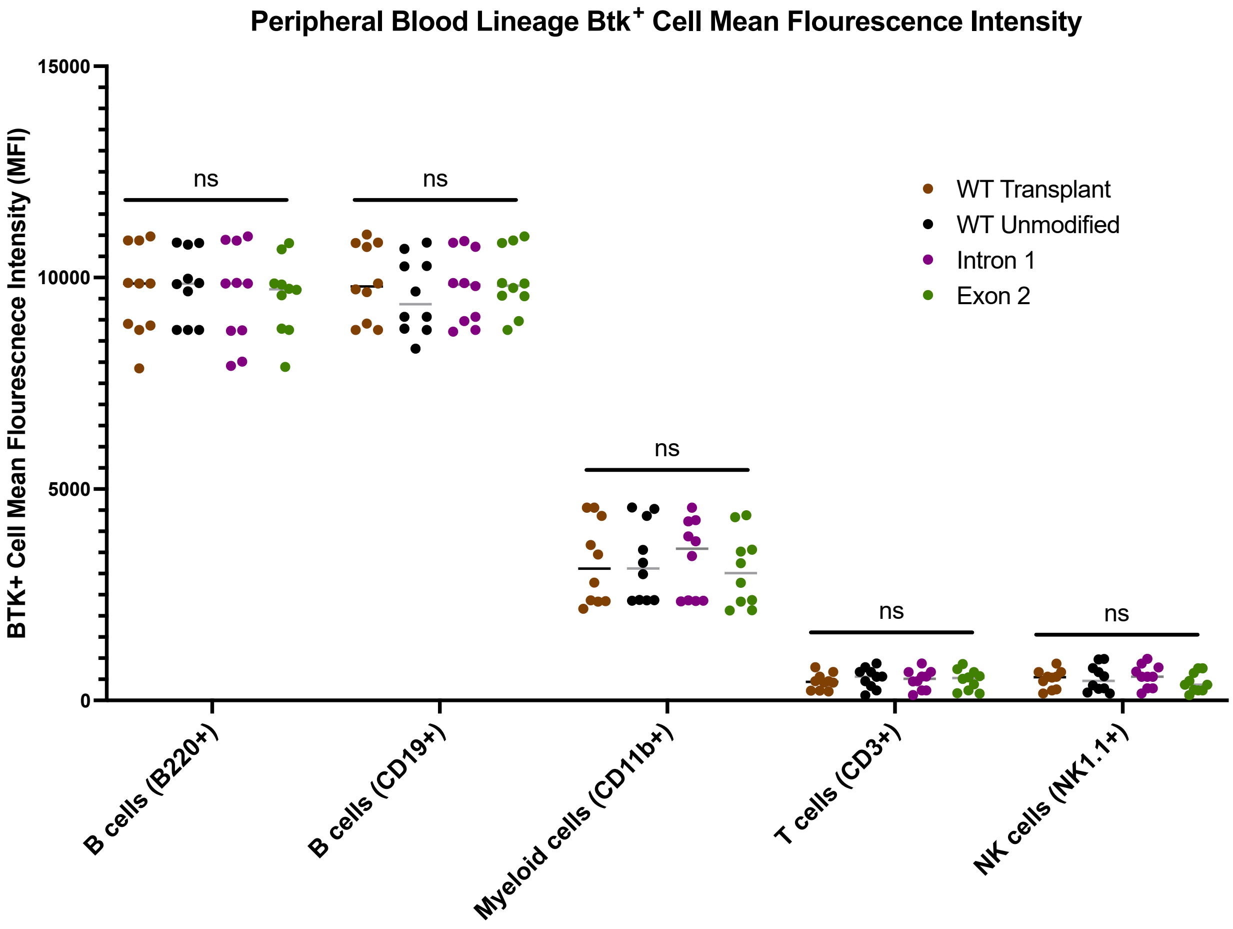

Figure S5: Peripheral Blood Lineage Btk$^+$ Cell MFI. Following extracellular staining of peripheral blood cells, intracellular flow cytometry was performed to determine both the percentage positive, and MFI of Btk expression in each immune cell lineage. Y-axis represents mean fluorescence intensity of Btk expression within each immune lineage.

Table S1: Statistical Analysis for figure 3B: Percentages of B-progenitors in the Bone Marrow. Table displaying Statistical significance and corresponding P-values for figure 3B. Comparisons are between intron 1/exon 2 versus WT transplant and Mock transplant conditions. Statistical analysis performed was two-way anova analysis combined with Tukey's multiple comparisons test.

Table S1: Statistical Analysis for figure 3B: Percentages of B-progenitors in the Bone Marrow		
Tukey's multiple comparisons test		
	**Summary**	**Adjusted P Value**
**Pro/Pre B-cells**		
Mock Transplant vs. Intron 1	****	<0.0001
Mock Transplant vs. Exon 2	****	<0.0001
WT Transplant vs. Intron 1	ns	0.9869
WT Transplant vs. Exon 2	ns	0.6507
**Immature B-cells**		
Mock Transplant vs. Intron 1	*	0.015
Mock Transplant vs. Exon 2	*	0.0346
WT Transplant vs. Intron 1	ns	0.9948
WT Transplant vs. Exon 2	ns	0.9646
**Mature B-cells**		
Mock Transplant vs. Intron 1	**	0.0014
Mock Transplant vs. Exon 2	*	0.0109
WT Transplant vs. Intron 1	ns	>0.9999
WT Transplant vs. Exon 2	ns	0.9817

Table S2: Statistical Analysis for figure 4B: Percentages of B-cells in the Spleen. Table displaying Statistical significance and corresponding P-values for figure 4B. Comparisons are between intron 1/exon 2 versus WT transplant and Mock transplant conditions. Statistical analysis performed was two-way anova analysis combined with Tukey's multiple comparisons test.

Table S2: Statistical Analysis for figure 4B: Percentages of B-cells in the spleen		
Tukey's multiple comparisons test		
	**Summary**	**Adjusted P Value**
**T1**		
Mock Transplant vs. Intron 1	ns	>0.9999
Mock Transplant vs. Exon 2	ns	>0.9999
WT Transplant vs. Intron 1	ns	0.9994
WT Transplant vs. Exon 2	ns	>0.9999
**T2**		
Mock Transplant vs. Intron 1	ns	>0.9999
Mock Transplant vs. Exon 2	ns	>0.9999
WT Transplant vs. Intron 1	ns	0.932
WT Transplant vs. Exon 2	ns	0.9043
**T3**		
Mock Transplant vs. Intron 1	ns	>0.9999
Mock Transplant vs. Exon 2	ns	>0.9999
WT Transplant vs. Intron 1	ns	>0.9999
WT Transplant vs. Exon 2	ns	>0.9999
**FO**		
Mock Transplant vs. Intron 1	ns	0.8047
Mock Transplant vs. Exon 2	ns	>0.9999
WT Transplant vs. Intron 1	ns	0.1166
WT Transplant vs. Exon 2	ns	0.944
**B1**		
Mock Transplant vs. Intron 1	ns	0.9986
Mock Transplant vs. Exon 2	ns	0.5663
WT Transplant vs. Intron 1	ns	0.2917
WT Transplant vs. Exon 2	ns	0.9993
**MZ**		
Mock Transplant vs. Intron 1	ns	0.5314
Mock Transplant vs. Exon 2	ns	0.5051
WT Transplant vs. Intron 1	ns	0.5018
WT Transplant vs. Exon 2	ns	0.4653

Table S3: Statistical Analysis for figure 5L: Low-Affinity NP-IgG1 Antibody Levels. Table displaying Statistical significance and corresponding P-values for figure 5L. Comparisons are between intron 1/exon 2 versus WT transplant and Mock transplant conditions. Statistical analysis performed was two-way anova analysis combined with Tukey's multiple comparisons test.

Table S3: Statistical Analysis for figure 5L: Low-Affinity NP-IgG1 Antibody Levels		
Tukey's multiple comparisons test		
	**Summary**	**Adjusted P Value**
**Day 0**		
Mock Transplant vs. Intron 1	ns	>0.9999
Mock Transplant vs. Exon 2	ns	>0.9999
WT Transplant vs. Intron 1	ns	>0.9999
WT Transplant vs. Exon 2	ns	>0.9999
**Primary**		
Mock Transplant vs. Intron 1	****	<0.0001
Mock Transplant vs. Exon 2	****	<0.0001
WT Transplant vs. Intron 1	ns	>0.9999
WT Transplant vs. Exon 2	ns	>0.9999
**Day 28**		
Mock Transplant vs. Intron 1	**	0.004
Mock Transplant vs. Exon 2	****	<0.0001
WT Transplant vs. Intron 1	ns	0.9971
WT Transplant vs. Exon 2	ns	0.8987
**Secondary**		
Mock Transplant vs. Intron 1	****	<0.0001
Mock Transplant vs. Exon 2	****	<0.0001
WT Transplant vs. Intron 1	ns	0.3501
WT Transplant vs. Exon 2	ns	0.8392

Table S4: Statistical Analysis for figure 5M: Low-Affinity NP-IgG1 Antibody Levels. Table displaying Statistical significance and corresponding P-values for figure 5L. Comparisons are between intron 1/exon 2 versus WT transplant and Mock transplant conditions. Statistical analysis performed was two-way anova analysis combined with Tukey's multiple comparisons test.

Table S4: Statistical Analysis for figure 5M: High-Affinity NP-IgG1 Antibody Levels		
Tukey's multiple comparisons test		
	**Summary**	**Adjusted P Value**
**Day 0**		
Mock Transplant vs. Intron 1	ns	>0.9999
Mock Transplant vs. Exon 2	ns	>0.9999
WT Transplant vs. Intron 1	ns	>0.9999
WT Transplant vs. Exon 2	ns	>0.9999
**Primary**		
Mock Transplant vs. Intron 1	*	0.0107
Mock Transplant vs. Exon 2	**	0.0065
WT Transplant vs. Intron 1	ns	0.9995
WT Transplant vs. Exon 2	ns	0.997
**Day 28**		
Mock Transplant vs. Intron 1	*	0.0399
Mock Transplant vs. Exon 2	*	0.0283
WT Transplant vs. Intron 1	ns	0.9436
WT Transplant vs. Exon 2	ns	0.9079
**Secondary**		
Mock Transplant vs. Intron 1	****	<0.0001
Mock Transplant vs. Exon 2	****	<0.0001
WT Transplant vs. Intron 1	ns	0.9956
WT Transplant vs. Exon 2	ns	0.7579
